# Metformin Acutely Mitigates Oxidative Stress in Human Atrial Tissue: A Pilot Study in Overweight Non-Diabetic Cardiac Patients

**DOI:** 10.3390/life12122058

**Published:** 2022-12-08

**Authors:** Ana Lascu, Loredana-Nicoleta Ionică, Adrian-Petru Merce, Maria-Daniela Dănilă, Lucian Petrescu, Adrian Sturza, Danina-Mirela Muntean, Caius Glad Streian

**Affiliations:** 1 Department III Functional Sciences—Pathophysiology, “Victor Babeş” University of Medicine and Pharmacy of Timișoara, E. Murgu Sq., No. 2, 300041 Timişoara, Romania; 2 Centre for Translational Research and Systems Medicine, “Victor Babeş” University of Medicine and Pharmacy of Timișoara, E. Murgu Sq., No. 2, 300041 Timişoara, Romania; 3 Institute for Cardiovascular Diseases of Timișoara, “Victor Babeş” University of Medicine and Pharmacy of Timișoara, G. Adam Str., No. 13A, 300310 Timișoara, Romania; 4 Doctoral School Medicine-Pharmacy, “Victor Babeş” University of Medicine and Pharmacy of Timișoara, E. Murgu Sq., No. 2, 300041 Timişoara, Romania; 5 Department VI Cardiology—Cardiovascular Surgery, “Victor Babeş” University of Medicine and Pharmacy of Timișoara, E. Murgu Sq., No. 2, 300041 Timişoara, Romania; 6 Advanced Research Center of the Institute for Cardiovascular Diseases, “Victor Babeş” University of Medicine and Pharmacy Timisoara, E. Murgu Square No. 2, 300041 Timişoara, Romania

**Keywords:** metformin, atrial tissue, oxidative stress, echocardiographic parameters, non-diabetic overweight cardiac patients

## Abstract

Metformin, the first-line drug in type 2 diabetes mellitus, elicits cardiovascular protection also in obese patients via pleiotropic effects, among which the anti-oxidant is one of the most investigated. The aim of the present study was to assess whether metformin can acutely mitigate oxidative stress in atrial tissue harvested from overweight non-diabetic patients. Right atrial appendage samples were harvested during open-heart surgery and used for the evaluation of reactive oxygen species (ROS) production by means of confocal microscopy (superoxide anion) and spectrophotometry (hydrogen peroxide). Experiments were performed after acute incubation with metformin (10 µM) in the presence vs. absence of angiotensin II (AII, 100 nM), lipopolysaccharide (LPS, 1 μg/mL), and high glucose (Gluc, 400 mg/dL). Stimulation with AII, LPS, and high Gluc increased ROS production. The magnitude of oxidative stress correlated with several echocardiographic parameters. Metformin applied in the lowest therapeutic concentration (10 µM) was able to decrease ROS generation in stimulated but also non-stimulated atrial samples. In conclusion, in a pilot group of overweight non-diabetic cardiac patients, acute incubation with metformin at a clinically relevant dose alleviated oxidative stress both in basal conditions and conditions that mimicked the activation of the renin–angiotensin–aldosterone system, acute inflammation, and uncontrolled hyperglycemia.

## 1. Introduction

Metformin, a biguanide synthetized at the beginning of the past century, is nowadays the first-line pharmacotherapy for type 2 diabetes mellitus (T2DM) due to its remarkable capability to control plasma glucose levels, primarily via decreased hepatic glucose production/output associated with enhanced insulin sensitivity and increased peripheral glucose uptake [1,2]. In the long run, the drug exerts pleiotropic elucidated effects far beyond glucose control [3], which are responsible for cardiovascular protection [4] and the anti-ageing properties [5] via partially elucidated mechanisms. Metformin has also emerged as a putative anti-obesity drug, as demonstrated by clinical [6] and animal studies [7].

Several randomized controlled trials that have been conducted in non-diabetic patients with atherosclerosis/high cardiovascular risk aimed at providing evidence for the long-term benefits of metformin; both positive or neutral effects on the cardiovascular outcome have been reported, as recently reviewed [8]. At variance, the cardiovascular effects of short-term administration of the biguanide in non-diabetic cardiac patients are by far less investigated. Thus, in a placebo-controlled, double-blind study (the MetCAB trial) performed on patients without diabetes who underwent on-pump coronary artery bypass graft (CABG) surgery, metformin was administered for 3 days before CABG and the effect of drug pretreatment on the limitation of cardiac injury (measurement of troponin level in dynamics) as primary endpoint was assessed. The authors concluded that short-term pretreatment with metformin did not reduce the periprocedural myocardial injury in non-diabetic patients undergoing elective CABG surgery [9].

Oxidative stress represents one of the most significant insults disrupting cardiomyocyte homeostasis that has been reported to occur in most cardiovascular pathologies [10]. In the setting of heart failure (HF), oxidative stress has been systematically reported to impair cardiac mechanics via myofibrillar oxidation [11] and loss of myofibrillar Ca^2+^-sensitivity [12], which leads to further aggravation of both systolic and diastolic function [13]. Currently, this complex clinical syndrome is regarded as a “systemic mitochondrial cytopathy” [14] since mitochondria are the major sites of reactive oxygen species (ROS) generation. There is now a large body of evidence that overproduction of ROS was effectively counteracted by metformin, yet the complexity of its anti-oxidant effect is far from being elucidated [15]. The group of Gerald Shulman has unequivocally demonstrated the redox-dependent mechanism of action of the drug at therapeutically relevant concentrations (i.e., in μM range), as recently described in an excellent review [16]. Metformin in chronic administration has been reported to activate the mitochondrial electron transport system (ETS) and increase ATP production, besides the oxidative stress limitation [17].

Data literature regarding the acute effect of micromolar doses of the biguanide on human samples are scarce. As such, the present pilot study was double aimed (i) to assess whether metformin applied in a clinical relevant concentration can acutely mitigate the oxidative stress in atrial tissue harvested from cardiac non-diabetic overweight patients, in the presence vs. absence of angiotensin II (AII), lipopolysaccharide (LPS) and high glucose (Gluc), respectively, and (ii) to investigate whether the magnitude of oxidative stress assessed in atrial samples correlates with atrial and/or ventricular echocardiographic parameters.

## 2. Materials and Methods

The study was conducted in accordance with the principles of the Declaration of Helsinki and the protocol was approved by the Committee for Research Ethics of “Victor Babeș” University of Medicine and Pharmacy of Timișoara, Romania (no. 04/28.02.2020 and 04p/17/12/2020). Written informed consent was obtained from all patients prior to surgery.

The study group included 20 non-diabetic cardiac patients (14 males and 6 females) who were overweight (BMI = 27.21 ± 0.96 kg/m^2^) and diagnosed with heart failure with mid-range ejection fraction (HFmrEF) according to the left ventricular ejection fraction (LVEF = 47.10 ± 1.59%). Fifteen (75%) patients were diagnosed with severe valvular lesions that required surgical intervention. Among these, 7 (35%) patients presented severe mitral regurgitation, 6 (30%) patients had severe aortic valve stenosis, 4 (20%) patients had severe tricuspid regurgitation, 2 (10%) patients had severe aortic regurgitation, and 1 (5%) patient had severe mitral stenosis. Eleven (55%) patients were diagnosed with severe coronary lesions. Regarding the type of surgery, 3 (15%) patients underwent aortic valve replacement, 1 (5%) patient had mitral valve replacement, 4 (20%) patients had mitral valve replacement and tricuspid valve plasty, 1 (5%) patient underwent aortic valve replacement and mitral valve annuloplasty, 5 (25%) patients underwent aorto-coronary bypass graft, 4 (20%) patients had aortic valve replacement and aortocoronary bypass graft, and 2 (10%) patients had aorto-coronary bypass surgery and mitral valve annuloplasty. Postoperative atrial fibrillation was documented in 3 (15%) patients and was converted to sinus rhythm with drug therapy (amiodarone). As for comorbidities, fourteen (70%) patients presented preoperative hypertension which was controlled (with anti-hypertensive medication, ACE inhibitors/sartans ± calcium channel blockers) in all cases and thirteen (65%) patients had dyslipidemia which was controlled (with statins) in most cases.

Atrial myocardium was sampled during open-heart surgery once cardiopulmonary bypass was established by resecting the tip of the right atrial appendage (approximately 20–30 mg). The atrial samples were further transferred to the laboratory for all the experimental procedures. Each sample was divided in 8 pieces which was randomly assigned to one of the following groups: (1) Control, (2) Control + Metfomin, (3) AII, (4) AII + Metformin, (5) LPS, (6) LPS + Metformin, (7) GLUC, and (8) GLUC + Metformin.

The characteristics of the included patients (*n* = 20) are listed in Table 1.

### 2.1. Organ Culture

Atrial tissue samples were cleaned and incubated for 12 h at 37 °C in a culture medium (containing 0.1% bovine serum albumin) in the presence or absence of AII (100 nM), LPS (1 μg/mL), and high Gluc (400 mg/dL) with or without metformin (10 µM). Subsequently, the tissue was embedded in Tissue-Tek for the superoxide assessment in confocal microscopy with dihydroethidium (DHE) or used for H_2_O_2_ assessment in spectrophotometry with the Ferrous Oxidation-Xylenol Orange (FOX) assay according to a method described in ref. [18].

### 2.2. Oxidative Stress Assessment with the Ferrous Oxidation-Xylenol Orange (FOX) Assay

Hydrogen peroxide production was assessed in human atrial tissue samples using the FOX assay (PeroxiDetect Kit, Sigma Aldrich-Merck, Darmstadt, Germany). The principle of the assay is that peroxides oxidize Fe^2+^ to Fe^3+^ ions at acidic pH. The Fe^3+^ ions will form a colored adduct with xylenol orange, which is quantified spectrophotometrically at 560 nm according to a method described in refs. [18,19,20].

### 2.3. Oxidative Stress Assessment Using DHE in Confocal Microscopy

Superoxide generation in human atrial samples was determined using the dihydroethidium (DHE) probe as previously described [18,20]. Briefly, samples were embedded in the optimal cutting temperature (OCT) compound and snap frozen. The frozen fragments were cut in 8 µm thick cryosections and put on glass slides. After 3 washes with phosphate buffered saline (PBS), 5 min each, the cryosections were incubated in the dark with DHE for 30 min at room temperature. Excess DHE was removed by 3 additional washes with PBS. The slides were mounted with Vectashield (Vector Laboratories) and immediately analyzed in confocal microscopy (laser scanning confocal microscope Olympus Fluoview FV1000, Olympus Corporation, Tokyo, Japan). Images were obtained using laser excitation at 488 nm. Image analysis was performed using the ImageJ software version 1.52t (NIH, USA).

### 2.4. Statistics

Statistical data processing was performed with the GraphPad Prism software version 9.3.1 (GraphPad, USA). Data are presented as mean ± SEM and were analyzed using one-way ANOVA. Pearson’s linear correlation was used to determine correlations between oxidative stress and echocardiographic parameters. Values of *p* < 0.05 were considered statistically significant.

## 3. Results

### 3.1. Metformin in Acute Administration Mitigates Oxidative Stress in Human Atrial Tissue

**Figure 1:**
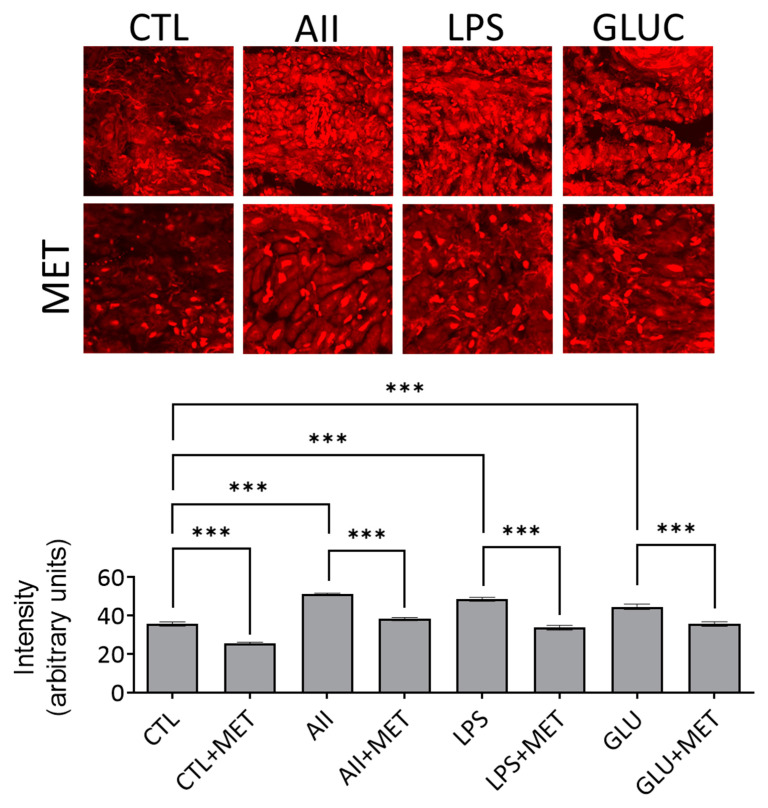
Metformin (MET) mitigated oxidative stress assessed by DHE staining in stimulated (AII, LPS, GLUC) and non-stimulated (CTL) atrial samples. *N* = 20, *** *p* < 0.001.

**Figure 2:**
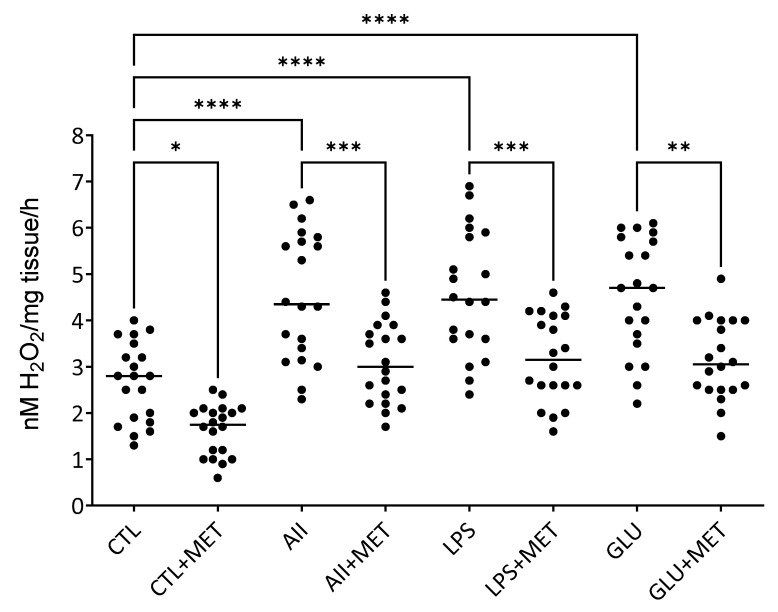
Metformin (MET) mitigated oxidative stress assessed by FOX assay in stimulated (AII, LPS, GLUC) and non-stimulated (CTL) atrial samples. *N* = 20, * *p* < 0.1, ** *p* < 0.01, *** *p* < 0.001, **** *p* < 0.0001.

Increased ROS generation is a central pathomechanism in cardiovascular pathology and metformin has been reported to elicit an anti-oxidant effect in chronic administration. In order to assess the acute anti-oxidant property of the biguanide in human samples, we investigated the effect of metformin on ROS generation in human atrial tissue after ex vivo stimulation (12 h) with AII (100 nM), LPS (1 μg/mL), and high glucose (400 mg/dL) and measured the ROS levels by two methods. As demonstrated by the DHE staining (Figure 1) and FOX assay (Figure 2), both superoxide anion and H_2_O_2_ generation were significantly increased in conditions that mimicked the activation of the renin–angiotensin–aldosterone system (RAAS), acute inflammation, and uncontrolled hyperglycemia, respectively. Importantly, the effect of metformin was also present in the control (CTL), non-stimulated samples incubated overnight with the drug, thus suggesting a potential beneficial role in the prevention of cardiovascular oxidative stress regardless of its magnitude (Figure 1 and Figure 2).

### 3.2. Atrial Oxidative Stress Correlates with Echocardiographic Parameters

**Figure 3:**
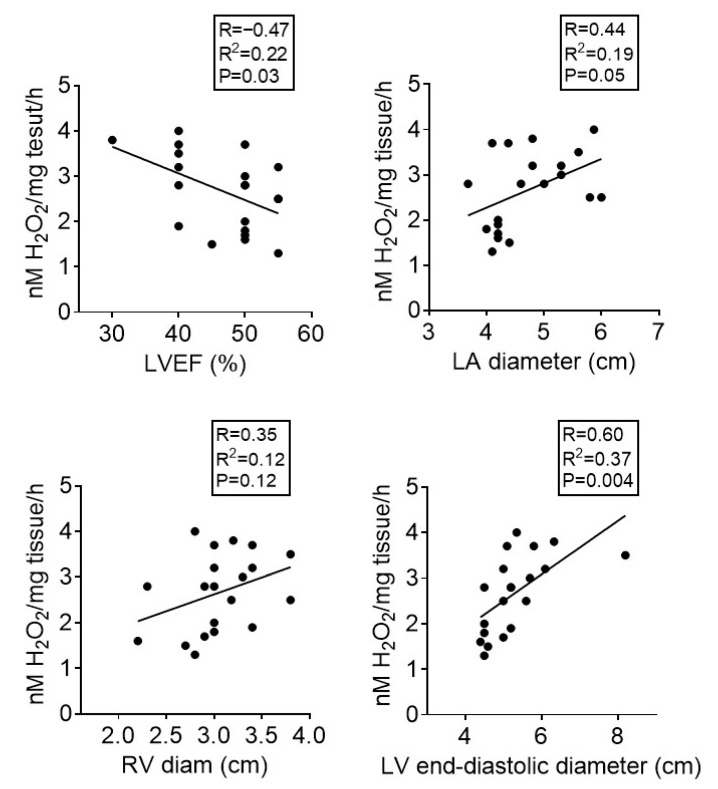
Correlation between ROS generation assessed by FOX technique and the echocardiographic parameters. LVEF—left ventricular ejection fraction; LA—left atrium; RV—right ventricle.

Correlation analysis was performed to evaluate a potential relationship between the echocardiographic parameters (LA diameter, LV end-diastolic diameter, and RV diameter) and the degree of oxidative stress (H_2_O_2_ measured by FOX assay) in the analyzed samples. We observed a positive correlation between the ROS levels and LA diameter, LV end-diastolic diameter, and RV diameter. At variance, a negative correlation was noticed between ROS and LVEF (Figure 3). As shown in Figure 3, most patients had mildly reduced or preserved EF, with one exception of HF with reduced EF. Of note, despite the fact that the H_2_O_2_ level was assessed in the right atrial appendages in both males and females with different cardiac pathologies requiring cardiac surgery, the above-mentioned correlations indirectly suggest that sex- and etiology-independent oxidative stress occurs in atria as well as in ventricles rather early in the evolution of heart failure in the case of overweight patients.

## 4. Discussion

Metformin (1,1-dimethylbiguanide), the cornerstone therapy for T2DM, has widely reported beneficial effects in numerous other pathologies such as cardiovascular, liver, and renal diseases as well as various types of cancer [21] via pleiotropic effects that have been recently covered in an excellent comprehensive review [22]. Despite being extensively studied for decades, the molecular mechanisms by which low-dose metformin exerts its glucose-lowering and life-extending effects have just started to become unraveled [23,24]. There is an unmet need for deciphering the role of metformin in non-diabetic individuals in order to broaden the scope of its indications [24]. Moreover, the results of several studies have been recently challenged due to the clinically irrelevant concentrations used in various in vivo and ex vivo experimental models [25].

We have here assessed the acute effects of the drug on both superoxide and hydrogen peroxide generation in human atrial samples harvested from overweight patients with no diabetes applied at 10 µM concentration (close to the steady-state plasma concentration) in conditions that mimic the renin–angiotensin–aldosterone system (RAAS) activation, acute inflammation, and uncontrolled hyperglycemia. The first important finding is the acute beneficial effect of the drug on the diseased atrial samples, including in basal conditions, at variance from its well known chronic cardiac protection.

Metformin has been earlier reported to decrease the AII-induced ROS generation in cardiac fibroblasts by inhibiting the activation of the PKC-NADPH oxidase pathway [26]. Of note, the acute administration of the drug has also been reported to elicit several protective effects in various non-cardiac experimental models. Thus, metformin elicited acute neuroprotection in a rat model of stroke induced by permanent middle cerebral artery occlusion [27]. Qu et al. reported that the same low dose (10 μM) of the drug elicited the resensitization of a multidrug-resistant MDA-MB-231 breast cancer cell line to several cytotoxic drugs [28]. Similarly, in a human promyelocytic cell line HL60, micromolar concentrations of metformin reactivated mitochondrial respiration [29]. Recently, metformin has been reported to enhance cell viability and proliferation in human HepaRG hepatocytes and to elicit in ovo vascular protection [30].

Plasma concentrations of metformin in long-term-treated humans range between ~1 µM and ~40 µM and the vast majority of in vitro studies aimed at elucidating its mechanisms of action have been performed with supratherapeutic drug concentrations [16,31]. Of note, in non-diabetic patients treated with 1 g metformin orally, the plasma concentration of the drug reached 25 µM within 3 h of administration [16]. Moreover, the in vivo relevance of its widely reported effect, i.e., inhibition of mitochondrial complex I of the electron transport chain and subsequent activation of AMP-activated protein kinase, has been recently challenged. Thus, an hormetic effect has been reported, with micromolar doses being able to activate the mitochondrial electron transport chain, increase ATP production, and limit oxidative stress, while millimolar concentrations were deleterious, inhibited complex I, and reduced the ATP cellular content [17].

Mitochondria are the main ROS sources, in particular when the ETS becomes dysfunctional and complex I can produce superoxide via both forward and reverse electron fluxes, depending on the substrates used. Metformin specifically decreased the superoxide generation driven by the reverse electron transfer but without increasing ROS generation through the forward direction [32]. However, the mechanisms behind the complex I inhibition are far from being elucidated, an intriguing finding being represented by the fact that concentrations required for inhibition are lower in intact cells than in isolated mitochondria and higher ex vivo as compared with the in vivo models [33]. The current paradigm postulates that the weak and reversible inhibition of complex I that results in a transient rise in intracellular AMP levels is crucial for the anti-hyperglycemic effect by inhibiting hepatic gluconeogenesis [34]. However, this classic paradigm has been recently challenged by the group of Gerald Shulman who proposed as novel mechanism of action for metformin, the complex IV-mediated inhibition of glycerol-3-phosphate dehydrogenase, which in turn reduced glycerol-related hepatic gluconeogenesis [35].

The observations that mitochondrial ROS production is central to the deleterious effects of hyperglycemia and that the protective properties of the drug administration are related to its mitochondrial effects have been reported more than two decades ago [36]. Thus, Hu et al. reported that metformin elicited direct protective effects against hyperglycemia and hypoxia/reoxygenation injury in H9C2 rat cardiomyoblasts via the activation of AMP-activated protein kinase (AMPK) and concomitant inhibition of Jun NH(2)-terminal kinase [37]. Metformin reduced ROS production and apoptosis in primary human and rat cardiomyocytes subjected to high-glucose stimulation in a protein phosphatase 2-dependent manner [38]. More recently, it has been demonstrated that metformin protected H9C2 cardiomyocytes against high glucose-induced elevated oxidative stress via a mechanism that involved stimulated mitochondrial biogenesis, as indicated by increased expression of several mitochondrial genes [39].

We report here the capability of metformin to decrease both the basal and stimulated ROS (superoxide and hydrogen peroxide) production in a pilot group of overweight cardiac patients with neither diagnostic nor treatment for diabetes. This observation is in line with our results that previously reported the effect of the same dose of metformin (10 μM, 12 h incubation) on cardiac samples harvested from rats with diet-induced obesity and prediabetes. In the animal model, the acute anti-oxidant effect was not related to a scavenger activity of the drug [20]. Importantly, the acute anti-oxidant effect of the drug was recapitulated when applied ex vivo in the same dose on aortic rings isolated from rats with diet-induced obesity [18] and also, on internal mammary arteries harvested from patients with coronary heart disease subjected to CABG (Lascu et al. submitted). These data are important because they confirm the acute cardiovascular protection provided by the biguanide in both animals and humans. In an early study performed on cultured human umbilical vein endothelial cells, Kukidome et al. reported that metformin normalized hyperglycemia-induced mtROS production by induction of MnSOD and promotion of mitochondrial biogenesis via AMPK activation [40].

Targeting mitochondrial dysfunction is nowadays an innovative therapeutic approach in the management of heart failure. A small double-blind, placebo-controlled, ascending-dose trial with elamipretide, a novel peptide which reduces the mitochondrial ROS production, acutely administered in patients with HF with reduced EF (≤35%) in a single 4 h infusion, demonstrated favorable changes in the left ventricular volumes that correlated with its peak plasma concentration [41]. Whether acute administration of metformin will be able to elicit such effect in the setting of heart failure has not been reported. We acknowledge as a study limitation the fact that the sources of oxidative stress targeted by metformin were not addressed. However, in the above-mentioned animal model of diet-induced obesity, we have demonstrated that overnight incubation with metformin (10 μM) decreased the expression of monoamine oxidase (MAO) in ventricular preparations isolated from obese rats with no effect in control samples. MAO is an enzyme with two isoforms (A and B) that constantly generates H_2_O_2_ at the outer mitochondrial membrane as ancillary product of the oxidative deamination of biogenic amines, mainly catecholamines in the heart. In heart samples harvested from the obese animals, metformin also mitigated the degree of oxidative stress as shown by both DHE staining and FOX assay. In order to assess whether the drug interferes with MAO expression in healthy animals, control samples were incubated overnight with serotonin (10 μM) in the presence vs absence of metformin and the amount of H_2_O_2_ was measured. Interestingly, the addition of the MAO substrate increased the H_2_O_2_ generation but metformin did not influence this process in the non-obese animals [20]. The acute anti-oxidant effect was also demonstrated in vascular preparations together with the decrease in MAO gene and protein levels and improvement in the endothelium-dependent relaxation of aortic rings isolated from the obese rats. We also reported that the enzymes responsible for catecholamine generation (MAO substrate), tyrosine hydroxylase, DOPA decarboxylase, and dopamine beta hydroxylase were upregulated in the vascular samples of the obese rats; however, incubation with metformin had no effect on their mRNA expression, suggesting that the decrease in the MAO expression is not related to the substrate availability [18]. In both heart and vascular samples, we also reported that metformin did not act as a ROS scavenger since it lacks the anti-oxidant capacity when compared to catalase, a classical ROS scavenger [18,20].

Oxidative stress is not only a promotor of cardiac and vascular damage but also a promotor of inflammation in the cardiovascular system [42]. Both oxidative stress and low-grade systemic inflammation are leading pathomechanisms behind the development of heart failure with preserved ejection fraction (HFpEF) [43]. They are both triggered, in particular, by the non-cardiac comorbidities, such as obesity and metabolic syndrome, that have been constantly reported to occur in patients with HFpEF [44].

In this pilot group of overweight patients with mid-range ejection fraction (HFmrEF), the second important finding is that the magnitude of oxidative stress assessed in the right atria positively correlated with the cavity changes in left atria and ventricles, with the highest correlation for the left ventricle. Since the correlation was also present with the ventricular echographic parameters, it is tempting to speculate that changes in the atria might be a relevant proxy for ventricular abnormalities in cardiac patients with HF in early stages. It has been recently reported that the life-course cumulative burden of BMI is positively and significantly associated with the development of left ventricular hypertrophy in midlife [45]. Whether this observation is true for oxidative stress is worth being investigated. The most significant correlation occurred between the oxidative stress and the left ventricle end-diastolic diameter. Additionally, the higher the H_2_O_2_ production, the lower the LV ejection fraction, suggesting that oxidative stress is likely to early contribute to the onset of contractile impairment in the evolution of heart failure. It has been reported a decade ago by the group of Fabio Di Lisa that oxidative changes of actin and tropomyosin were significantly increased in LV biopsies from explanted hearts harvested from patients with end-stage HF (NYHA class IV) and were inversely correlated to LVEF [46]. Currently, it is widely accepted that mitochondria are both sources and targets of the oxidative modifications that contribute to the progression of heart failure [47]. However, the contribution of individual enzymatic (and non-enzymatic) ROS sources in patients with HF lacking obesity or diabetes is far from being elucidated. Assessment of the oxidative stress in the diseased atrial samples (which was decreased by metformin) and the correlation of its magnitude with atrial and ventricular size/impaired function in patients with either valvular or coronary artery diseases are important observations that are worth further exploring in larger clinical trials.

Early mitigation of acute oxidative stress represents an unmet therapeutic goal in the setting of heart failure [48], particularly when related to cardiopulmonary bypass in patients undergoing open heart surgery, and the anti-diabetic therapy is nowadays the best candidate for drug repurposing [49]. While several mechanistic evidence are available to explain the long-term favorable actions of metformin in heart failure [50], the largely unexplored effects of this old drug in acute administration requires a closer look in order to decipher the molecular mechanisms underlying cardioprotection.

## 5. Conclusions

Metformin in a clinically relevant concentration mitigated oxidative stress in human atrial samples harvested from overweight non-diabetic patients undergoing revascularization therapy. The magnitude of oxidative stress positively correlated with the LA diameter, LV end-diastolic diameter, and RV diameter and negatively with the LVEF. Whether the acute administration of the biguanide prior to open-heart surgery could elicit beneficial effects on cardiovascular outcomes warrants further investigation.

## Figures and Tables

**Table 1:** Characteristics of the study group.

Parameter	Value
Age (years)	65.35 ± 1.7
Sex (M/F)	14/6
BMI (kg/m^2^)	27.21 ± 0.96
Systolic Blood Pressure (mmHg)	128.85 ± 3
Diastolic Blood Pressure (mmHg)	76.1 ± 1.9
Heart Rate (b/min)	67.55 ± 2.02
Erythrocyte Sedimentation Rate (mm/h)	23.65 ± 4.56
Red Blood Count (mil./mm^3^)	4.55 ± 0.14
PCV (%)	41.63 ± 1.31
Hemoglobin (g/dL)	13.84 ± 0.42
White Blood Count (×10^3^/mm^3^)	7.66 ± 0.39
Platelets (×10^3^/mm^3^)	223.2 ± 12.18
Creatinine (mg/dL)	1.03 ± 0.07
Uric Acid (mg/dL)	7.73 ± 1.38
Total Cholesterol (mg/dL)	176.7 ± 12.85
HDL-Cholesterol (mg/dL)	41 ± 4.59
LDL-Cholesterol (mg/dL)	99.55 ± 16.19
Triglycerides	185 ± 53.57
FPG (mg/dL)	103.8 ± 4.29
ALAT (U/L)	23.95 ± 3.29
ASAT (U/L)	21.7 ± 1.73
Na^+^ (mmol/L)	140.9 ± 0.91
K^+^ (mmol/L)	4.18 ± 0.07
LA diameter (cm)	4.75 ± 0.16
LV end-diastolic diameter (cm)	5.28 ± 0.19
RV diameter (cm)	3.05 ± 0.09
LVEF (%)	47.10 ± 1.59

BMI—body mass index; PCV—packed cell volume; FPG—fasting plasma glucose; ALAT—alanine aminotransferase; ASAT—aspartate aminotransferase; LA—left atrium; LV—left ventricle; RV—right ventricle; LVEF—left ventricular ejection fraction.

## Data Availability

The authors confirm that the data supporting the findings of this study are available within the article.
